# Laboratory Investigation of Aging Resistance for Rubberized Bitumen Modified by Using Microwave Activation Crumb Rubber and Different Modifiers

**DOI:** 10.3390/ma13194230

**Published:** 2020-09-23

**Authors:** Ben Zhang, Huaxin Chen, Honggang Zhang, Yongchang Wu, Dongliang Kuang, Fengjun Guo

**Affiliations:** 1 School of Materials Science and Engineering, Chang’an University, Xi’an 710064, China; 2015031003@chd.edu.cn (B.Z.); 2015031001@chd.edu.cn (Y.W.); kuangdl@chd.edu.cn (D.K.); 2 Guangxi Transportation Research & Consulting Co., Ltd., Nanning 530000, China; zhang_paper0@163.com; 3 Shanxi Province Expressway Group Limited Liability Company, Taiyuan 030006, China; guo_eg@163.com

**Keywords:** rubberized bitumen, microwave-pretreated, warm mix additive, trans-polyoctenamer, aging resistance

## Abstract

Different modification methods, such as adding modifiers and pretreating crumb rubber, have been developed to achieve decent engineering properties and reduce the viscosity of rubberized bitumen. This study evaluated the influence of the modification methods on the aging resistance for rubberized bitumen. Two types of crumb rubber—a 40-mesh crumb rubber and a microwave-pretreated crumb rubber—and two kinds of modifiers—Sasobit and Trans-polyoctenamer—were selected to prepare rubberized bitumen. The samples were subjected to a Thin-Film Oven Test for the simulation of the short-term aging condition, while a Pressure-Aging-Vessel test was used to simulate the long-term aging condition. The indexes of rubberized bitumen, including softening point, elastic recovery ratio, maximum load, ductility, fracture energy, phase angle, and dynamic modulus, were tested before and after aging. The result showed that trans-polyoctenamer displayed the best resistance to short-term aging, while Sasobit significantly improved the fracture energy of rubberized bitumen after short-term aging. Microwave pretreated partially destroyed the internal structure of crumb rubber, leading to a decrease of short-term aging resistance for rubberized bitumen. Compared with short-term aging, the changing trends of various indexes were basically same, except the discrepancy of properties indexes was reduced after long-term aging.

## 1. Introduction

Rubberized bitumen was extensively used in the bitumen pavement because of its excellent performance [1,2]. It has been proved that crumb rubber (CR) effectively improved the resistance to the rutting, low temperature and fatigue cracking, and thermal oxygen aging of pavement [3,4]. However, the differences of density and polarity are the main causes for incompatibility between CR and bitumen, which results in the lack of storage stability. The swelling of CR also leads to an excessively high viscosity of rubberized bitumen. To resolve the limitation of rubberized bitumen, different modification methods have been proposed to reduce the incompatibility and high viscosity of rubberized bitumen [5,6,7,8,9,10]. Warm mix additives (WMA) and pre-degradation CR were two effect methods to decrease the high viscosity of rubberized bitumen. Another method is to add reactive modifiers such as trans-polyoctenamer (TOR), the purpose of which is to improve the compatibility between CR and bitumen [11]. Previous studies proved that the effects of these methods on the compatibility between CR and bitumen were effective, while few studies focused on the aging resistance for rubberized bitumen prepared with these methods.

To ensure the workability of bituminous mixtures, the mixing and compaction temperatures need to exceed 160 °C, creating the volatilization and light components oxidation (short-term aging (STA)). The oxidation, steric hardening, and ultraviolet radiation will gradually conduct under the vehicle loads and environmental impact during the pavement in service (long-term aging (LTA)) [12,13]. The incorporation of CR to bitumen transforms rubberized bitumen to a solid–liquid two-phase system at high temperature, which significantly complicates the aging process of bitumen [14,15]. The aging of bitumen, continuous swelling, and the degradation behavior of CR determine the aging process of rubberized bitumen [16]. Therefore, multiple factors have great influence on the aging resistance of rubberized bitumen such as aging conditions, CR content, CR particle size, and source of base bitumen [17]. It has been proved that CR was an efficient modification, which effectively improved the resistance for the aging process of bitumen [3,18,19]. Lee et al. [12] found that the light components were earlier absorbed by CR from the bitumen, which helped reduce the rate of asphaltenes formation. According to the Thermogravimetry-Fourier Transform Infrared test, it has been proved that the CR could significantly reduce the amount of released volatiles for the bitumen, and this phenomenon could improve the aging resistance of bitumen [20]. Moreover, the components introduced by the dissolution of CR can offset the light components volatilization during STA [3]. Wang et al. [13] investigated the effects of laboratory STA and LTA on the chemistry and rheology of rubberized bitumen, and the results showed that CR improved the aging resistance of rubberized, which reflected by the decreased carbonyl and sulfoxide indices. Moreover, they found that a higher CR content could improve the aging resistance of rubberized bitumen. In addition, carbon black and zinc oxide contained in CR can also act as an antioxidant to mitigate the aging happening in rubberized bitumen [21].

Excessively high viscosity leads to a higher operating temperature during rubberized bitumen mixture production, which makes the STA behavior of binders more important. WMA is an effective way to decrease the viscosity of rubberized bitumen, while due to lower viscosity, the processing and compaction temperature of bitumen mixtures will decrease significantly [22,23]. Some researchers indicated that WMA effectively improved the rutting resistance after the aging process, and the Sasobit-modified mixture showed the highest load cycle among the mixtures [24,25].

It has also been found that enhancing chemical bonding could improve the compatibility of rubberized bitumen [4,5,6,7,9]. Various crosslinking agents have been developed to enhance the interfacial strength between CR and bitumen, among which TOR reveals efficient modification effect [26,27,28,29,30]. It has been noted that the properties of rubberized bitumen such as elastic recovery, high-temperature stability, and modulus were improved by the use of TOR [4]. In addition, it also improved aging resistance effectively [27,31]. However, Liang et al. [7] indicated that TOR-modified rubberized bitumen hardens obviously because of the aging process.

The state of CR’s vulcanization directly affects the degradation and swelling of CR in bitumen. Short-term microwave activation can result in the desulfurization and degradation of CR, which accelerates the reaction course between CR and bitumen. It has been proved that bitumen modified by microwave pretreatment CR had a lower viscosity [32,33,34,35]. Xiaolong Yang [20] determined that CR modified by microwave activation had a more efficient swelling reaction than CR. The recoverability of microwave-activated rubberized bitumen was improved after aging [36]. However, the desulfurization and degradation give rise to the attenuation of CR’s elastic property, which decreased the resistance to fatigue cracking for rubberized bitumen after aging.

As the most common methods applied in the rubberized bitumen preparation, the author had evaluated the influence of the modification methods on the physical and rheological properties for rubberized bitumen in the previous study [11]. The result showed that the modification methods had significant influences on the rubberized bitumen properties. In consideration of few studies focus on the influence of different modification methods on the aging property of rubberized bitumen at an identical preparation process. In this study, six kinds of rubberized bitumen samples were prepared under high temperatures and high shear rates. The Thin-Film Oven Test (TFOT) and Pressure-Aging-Vessel (PAV) test were used to simulate aging conditions. The comparison of various indexes before and after aging was discussed to evaluate the aging resistance of rubberized bitumen.

## 2. Objective and Approach

The main objective of this study is to evaluate the influence of different modification methods on the aging resistance of rubberized bitumen with an identical preparation process. The following subtasks were implemented to achieve the objective of this study.

◆ The TFOT and PAV test were used for the simulations of the aging conditions.
◆ Indexes including softening point, elastic recovery ratio, maximum load, ductility, and fracture energy were tested before aging, and after STA and LTA to evaluate the physical properties of rubberized bitumen.
◆ Dynamic shear rheometer (DSR) tests were conducted on rubberized bitumen samples (before aging, subjected to TFOT, and subjected to TFOT + PAV) to obtain the indexes including phase angle and dynamic modulus.

**Figure 1:**
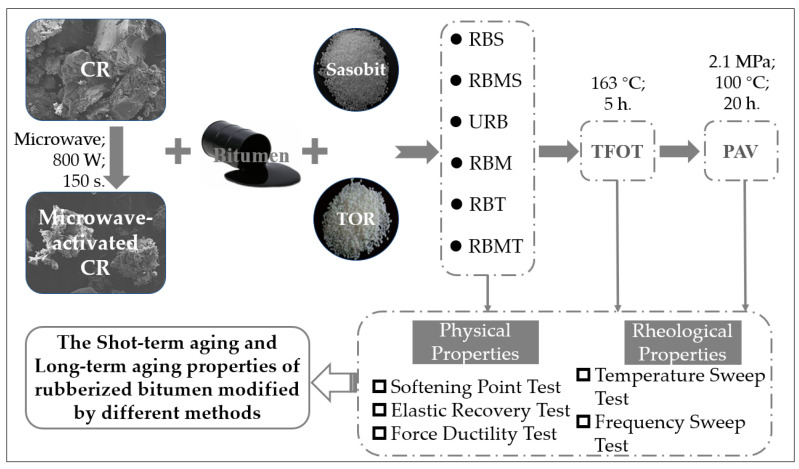
Flowchart of research steps in this study.

The differences of the indexes before aging, and after STA and LTA were compared and analyzed to evaluate the effect of the modification methods on the aging resistance of rubberized bitumen. The flowchart of research steps is presented in Figure 1.

## 3. Experimental

### 3.1. Materials and Samples Preparation

**Figure 2:**
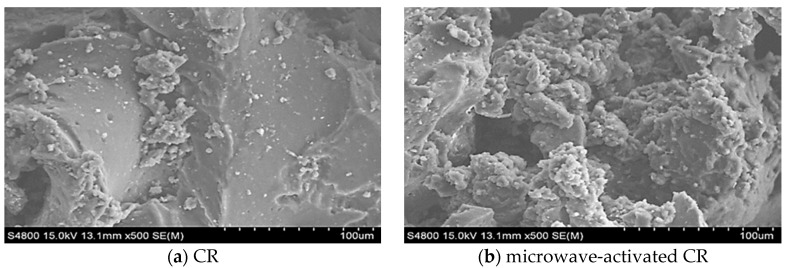
SEM image of crumb rubber (CR) particles and microwave-activated CR particles.

Table 1 presents the physical properties of SK#90 bitumen. CR was supplied by Xinyuda Environmental Protection Technology Co., Ltd., Wuwei, China. A household microwave oven (Midea Co. Ltd., Shunde, China) was used to apply microwave radiation to the rubber. The dried and dehydrated CR was subjected to microwave oven under a power of 800 W for 150 s. The microstructures of CR and pretreated CR are shown in Figure 2. As can be seen from Figure 2, the surface of CR is relatively flat and smooth. After microwave irradiation, the surface of CR became porous and uneven. This phenomenon can increase the reaction area and promote the swelling of CR in bitumen [11].

TOR is a solid polymer with a double bond structure that can crosslink the sulfur of the asphaltene and the sulfur on the CR’s surface to form a ring and mesh structure composed of chain polymers [7]. It was supplied by EVONIK Company and its application rate was 4.5% by weight of CR.

The WMA used in this research was Sasobit, which was supplied by Sasol—Wax Co., Ltd., Johannesburg, South Africa. The application rate of Sasobit was 1.0% by weight of bitumen. It is a granular and opaque pellet polymer, which has long chain aliphatic hydrocarbon obtained from coal gasification [37].

The six rubberized bitumens used in this study adopted the same preparation process. The details and labels of samples are shown in Table 2. CR and modifiers were gradually added to bitumen at a certain temperature and blended under high shear condition. The specific processes are given as follows.

(1) Blend the CR (or pre-treated CR) modifiers with the virgin bitumen previously conditioned at 150 °C by a 300 rpm stirring for 30 min.
(2) Shear the blended mixture at 4000 rpm and 200 °C for 60 min.
(3) Stir the blended mixture at 300 rpm and 180 °C for 60 min.
(4) Condition the prepared rubberized bitumen at 140 °C for 30 min.

### 3.2. Aging Methods

The TFOT at 163 °C for 5 h was used to simulate the STA of rubberized bitumen during bitumen mixture mixing, transportation, and paving progress, while the LTA samples were obtained by PAV tests for 20 h at 100 °C and 2.1 MPa [38]. In addition, the samples used in PAV test were aged by TFOT at 163 °C for 5 h first.

### 3.3. Analysis Methods

The softening point test and elastic recovery test were conducted in accordance with ASTM D36 and ASTM D6084, respectively [39,40]. The recovery test was conducted at 25 °C. The force ductility test (FDT) was conducted at 5 °C by using the samples apply for elastic recovery test. The complex shear modulus (G*) and phase angle (*σ*) were measured through temperature sweep test and frequency sweep test in accordance with ASTM D7175 [41]. The details of the experimental settings are shown in Table 3.

## 4. Results and Analysis

### 4.1. Physical Properties

#### 4.1.1. Softening Point

**Figure 3:**
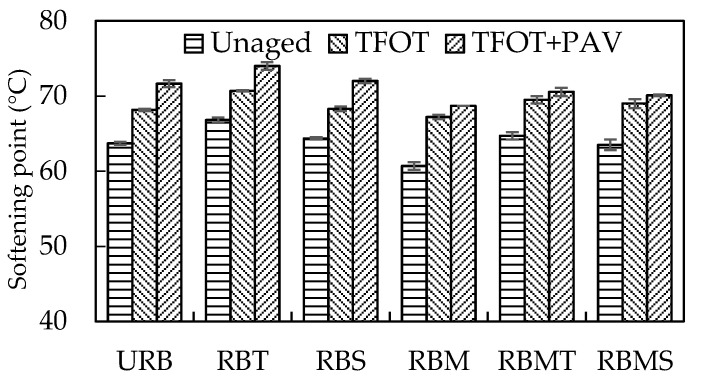
Effect of modification methods on the softening point of rubberized bitumen.

The effect of aging on the high-temperature stability of rubberized bitumen can be evaluated with softening point, which is also suggested to relate to the interaction between CR and bitumen [42]. Figure 3 shows the softening points of different samples before and after STA and LTA. The softening point of RBT was the highest before the aging process. It has reported that TOR could promote the distribution of CR, which increased the high-temperature performance of rubberized bitumen [9]. The unaged RBM showed the lowest softening point, as the microwave destroyed the vulcanization of CR partially. The softening point of unaged RBS improved slightly compared with unaged URB. In addition, for all samples, the softening point increased after aging to varying degrees. Liu [43] indicated that the softening point increment could be an effective index to evaluate the aging resistance of rubberized bitumen. Rubberized bitumen has a smaller softening point increment indicating an excellent resistance to the high temperature deformation. It was calculated following the equations below,

(1) $$∆T_{1}=T_{2}-T_{1}$$

(2) $$∆T_{2}=T_{3}-T_{1}$$

where *T*_1_, *T*_2_, and *T*_3_ are average softening point before aging, after 5 h TFOT, and after 5 h TFOT + 20 h PAV, respectively.

**Figure 4:**
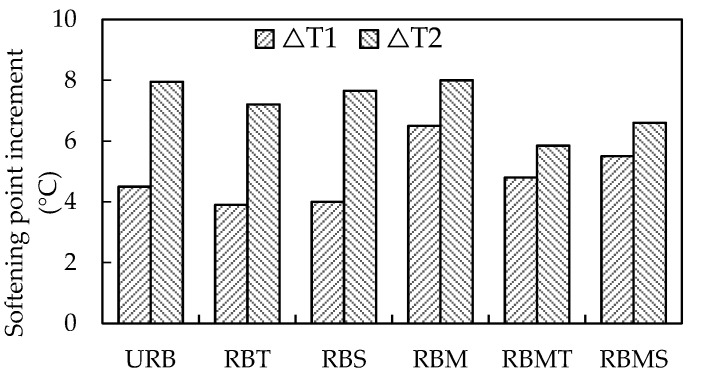
Effect of modification methods on the softening point increment of rubberized bitumen.

The results of softening point increment are shown in Figure 4, in which the RBT was the smallest among the results of ∆*T*_1_, followed by RBS and RB. The rubberized bitumen contained CR with 40-mesh size had a smaller ∆*T*_1_ compared with the rubberized bitumen contained CR activated by microwave. During the STA at 163 °C, the swelling reaction and degradation of CR will gradually advance [36,44]. Therefore, the main reactions happening in STA can be divided into the aging of bitumen and the swelling and degradation of CR. The microwave irradiation broke down the external and internal chemical bonds of the CR partially [7,11]. The destruction of chemical bond accelerated the desulfurization and degradation of CR particles, and the light component in the bitumen absorbed by the swelling action was released partly. This process weakened the aging resistance of rubberized bitumen, which resulted in a higher softening point increment of RBM.

According to Figure 4, the softening point increment of rubberized bitumen had a further increase after LTA. RBM had the highest ∆*T*_2_, and RBMT showed the lowest ∆*T*_2_ after LTA. Compared with URB, the addition of modifiers reduced the softening point increment of rubberized bitumen after LTA. ∆*T*_2_ of URB and RBM was impaired more heavily, indicating there relatively poor performance in LTA. RBMT and RBMS showed lower ∆*T*_2_, and this indicated that the combined effect of microwave activation and the modifiers had a favorable influence on the LTA.

#### 4.1.2. Elastic Recovery Ratio

**Figure 5:**
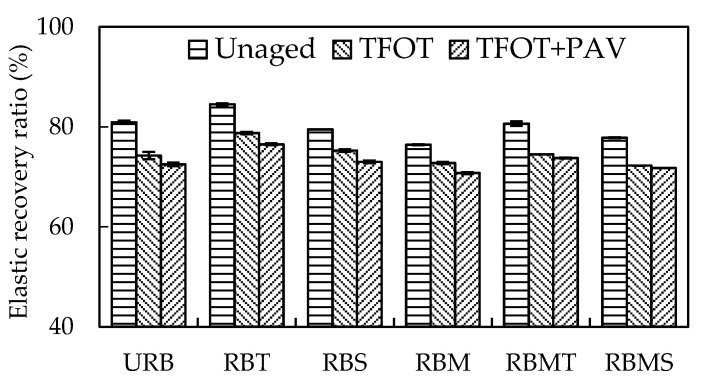
Effect of modification methods on the elastic recovery ratio of rubberized bitumen.

Figure 5 presents the effects of STA and LTA on the elastic recovery ratio of different rubberized bitumens. As can be seen, the elastic recovery ratio decreased obviously after LTA. RBT still had the highest elastic recovery ratio after STA, while RBM showed the lowest elastic recovery ratio. This behavior was consistent with the unaged samples. It has been proved that virgin bitumen was viscous and barely exhibited recovery at 25 °C [30]. The presence of CR with higher elasticity endows the rubberized bitumen with resilience. The STA generally stiffened the bitumen because of the components and the volatilization of light fractions [3]. The hardened bitumen made the CR particles face a greater resistance when its recover.

After LTA, the elastic recovery ratio of different rubberized bitumens decreased consistently. However, the magnitude of the decline was significantly reduced. It was because LTA conditions had a weak effect on the elasticity of CR particles. RBT had the highest elastic recovery ratio after LTA. TOR can effectively enhance the incorporation between CR and bitumen, and it stimulated rubberized bitumen to form an elastic structure. RBS had a similar elastic recovery ratio with URB whether it was unaged binder or after STA and LTA, which may be attributable to limit interaction between Sasobit and CR.

#### 4.1.3. Force Ductility

**Figure 6:**
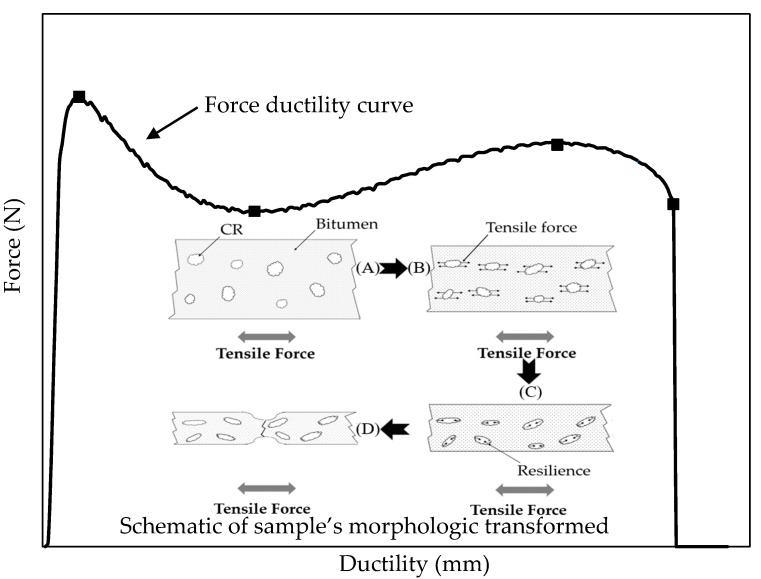
The force–ductility curve of rubberized bitumen.

Numerous studies have asserted that the cohesive strength of polymer-modified bitumen could be evaluated by FDT effectively [3,45,46]. The typical force–ductility curve of rubberized bitumen is shown in Figure 6. One of the biggest differences between rubberized bitumen and other binders is that it will present two yielding points in the force–ductility curve of rubberized bitumen. This phenomenon is associated with the elastic recovery ability of CR [3,47]. It can be seen that the force and ductility show a linear relationship until the maximum load (*F_max_*) is attained. In this region, the force is determined by the cohesive strength of bitumen (Figure 6A) [45,48]. The force declines after reaching *F_max_*, and this phenomenon is due to the flow of bitumen and slight deformation of CR (Figure 6B). Thereafter, the deformation of CR reaches a certain level, and the resilience of CR reacts to bitumen, resulting in a slight increase in force (Figure 6C) [46,49]. Eventually, the bitumen was fractured and reached failure ductility (Figure 6D). Indexes including *F_max_*, ductility, and fracture energy (*W*) were analyzed to evaluate the cohesive strength of rubberized bitumen.

**Figure 7:**
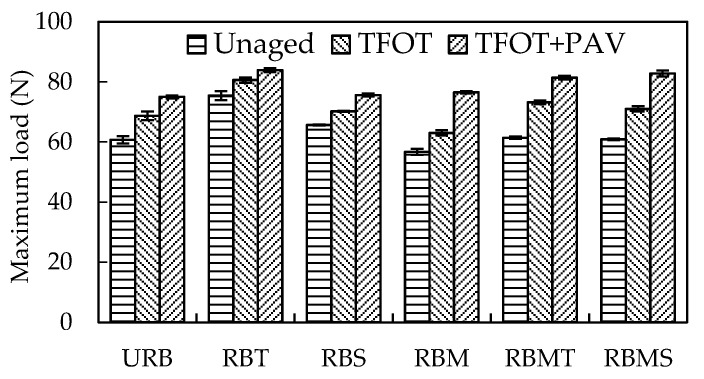
Effect of modification methods on the *F_max_* of rubberized bitumen.

**Figure 8:**
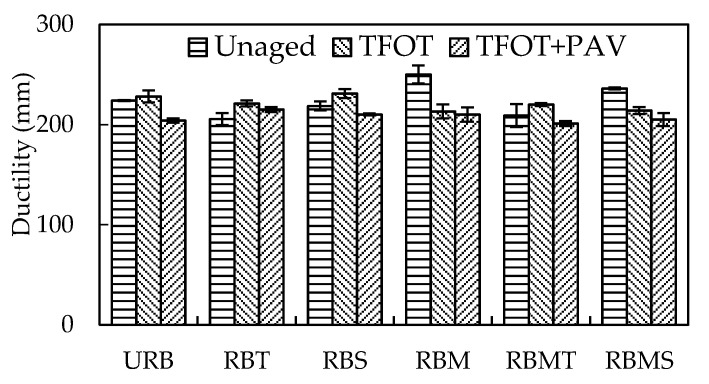
Effect of modification methods on the ductility of rubberized bitumen.

The *F_max_* and ductility of rubberized bitumen before and after aging are shown in Figure 7 and Figure 8. For the unaged rubberized bitumen, the ductility of RBM was largest and its *F_max_* was the lowest. This can be attributed to the desulfurization and depolymerization of CR, which can generate light components and modify the ductility of the binder. The ductility of RBT was the lowest and its *F_max_* was the highest. TOR had limited effect on the desulfurization and depolymerization of CR, and its main function was to link the CR surface and binders. The ductility of RBS was close to URB, and the difference was 5.5 mm. Sasobit primarily modified the molecular structure of free bitumen at high temperature, and the influence on the flexibility of rubberized bitumen at low temperature was influenced by the properties of CR and its reaction process with bitumen [11].

After STA, the *F_max_* of different rubberized bitumens increased to various degrees. However, the ductility of different rubberized bitumens had no consistent trend with *F_max_*. The ductility of RBM and RBMS decreased after STA, and the others tended to increase. This phenomenon may be explained by the swelling and degradation of CR in aging process. Swelling can excite the elasticity of CR, while the degradation of CR can supplement the light component volatilized in the aging process. The constituents of matrix bitumen are volatilized and reconstituted during STA, which led to the stiffness of bitumen. *F_max_* was mainly affected by the hardness of the bitumen.

It also can be seen from Figure 7 and Figure 8 that *F_max_* of different rubberized bitumens increased and their ductility decreased after LTA. LTA transforms the bitumen into a stiffer binder. Therefore, the variation tendency of the *F_max_* and ductility of rubberized bitumen was opposite. In addition, it was interesting that the results of ductility had only a small difference. The *F_max_* of rubberized bitumen contained microwave activation; CR had a more obvious increase compared with that of rubberized bitumen containing untreated CR.

**Figure 9:**
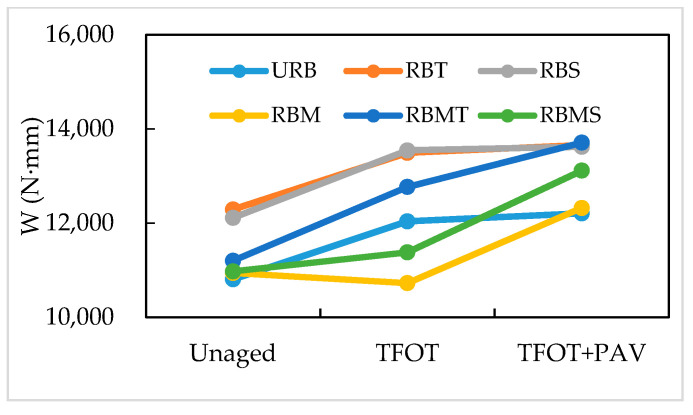
Effect of modification methods on the fracture energy of rubberized bitumen.

Figure 9 presents the *W* of rubberized bitumens. The inconsistency of *W* is associated with the swelling and disintegration of CR, and the aging stages of bitumen [3]. As shown in Figure 9, besides RBM, the *W* of rubberized bitumens increased after STA. Further, all samples had enhanced *W* after LTA compared with unaged binders. After STA, RBT and RBS showed a higher *W* and RBM showed the lowest *W*. After LTA, RBM showed a lower *W* than URB. Compared with the results of *W* after STA, the *W* of rubberized bitumens increased after LTA, except URB.

### 4.2. Rheological Properties

#### 4.2.1. Temperature Sweep Test

**Figure 10:**
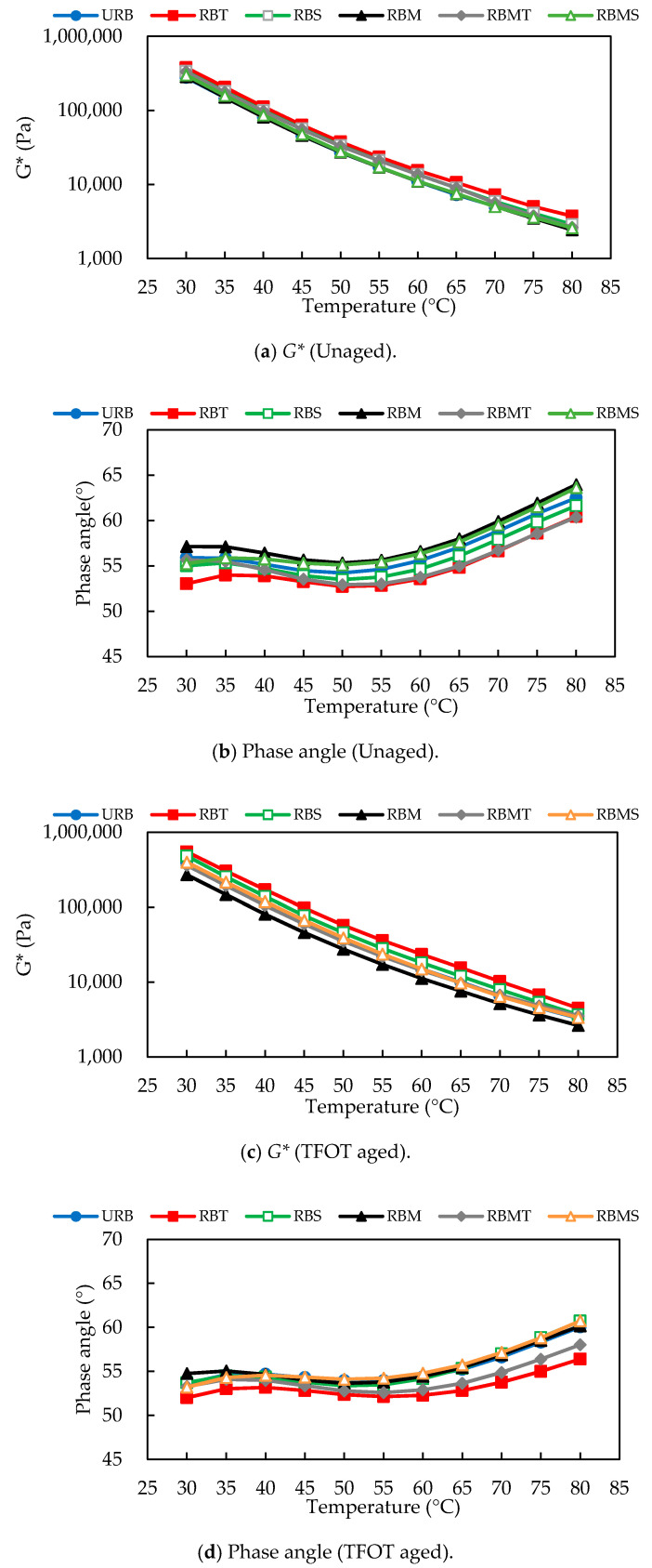

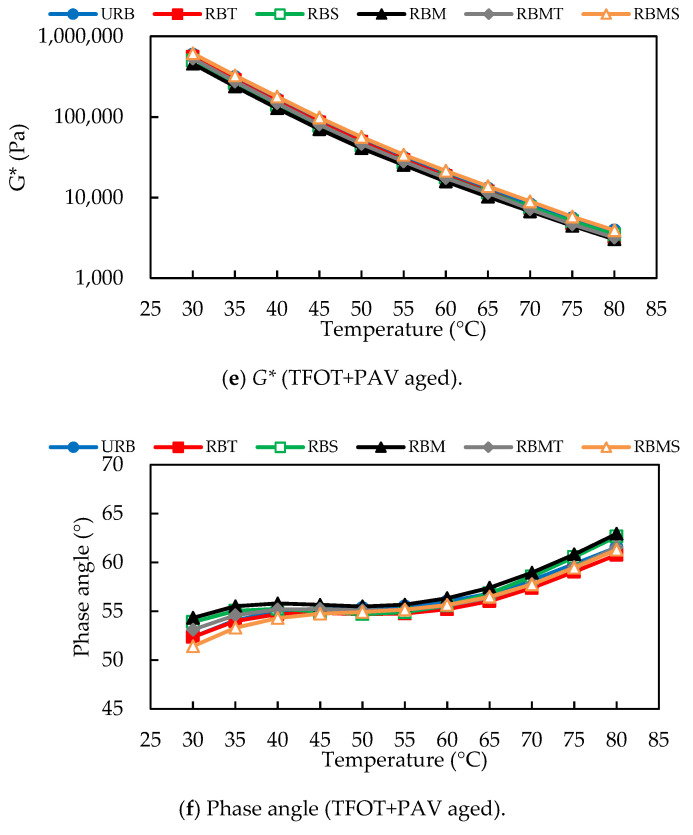
Effect of modification methods on the complex modulus and phase angles of rubberized bitumen depends on temperature sweep test.

The temperature sweep test of rubberized bitumen before and after aging was tested by DSR. According to Figure 10, the phase angles of the unaged binders decreased slightly before the temperature at 50 °C, and then increased with the temperature rising. From Figure 10d, after STA, the phase angle curves presented a same trend with the unaged samples. However, the difference between the phase angle curves was reduced. Moreover, the rising slope of the curve slowed down. In Figure 10f, phase angle generally increased with the temperature rising after LTA, and a plateau existed in the phase angle curves. The difference between the phase angle curves further decreased.

For the unaged rubberized bitumen, the binders modified by TOR had a smaller phase angle than others. RBM shown the largest phase angle, which could be explained by the degradation and desulfurization of CR. RBS shown a lower phase angle than URB, but the extent of the improvement was insignificant.

After STA, RBT, and RBMT still maintained small phase angles, but the difference between them increased. RBS had a lower phase angle than URB from 30 °C to 65 °C, but when the temperature beyond 65 °C, the phase angle of RBS was gradually larger than URB. This phenomenon may relate to the change of rheological property of Sasobit with temperature.

After LTA, RBT displayed the lowest phase angle and RBM showed the highest, but the difference between them significantly reduced. In Figure 10a,c,e, the regularity of difference of G* between six binders was basically uniform and consistent with the phase angle. As anticipated, RBT and RBM displayed the highest and lowest G* respectively before and after aging.

#### 4.2.2. Frequency Sweep Test

**Figure 11:**
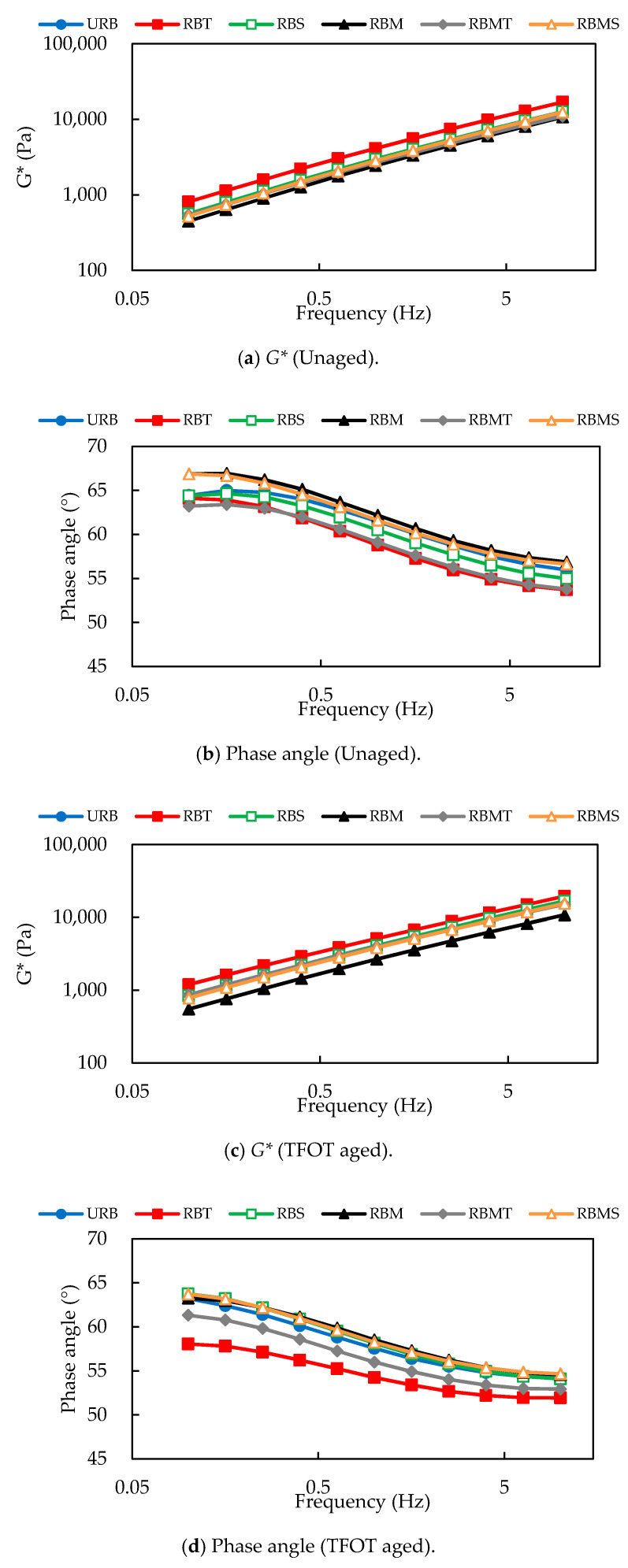

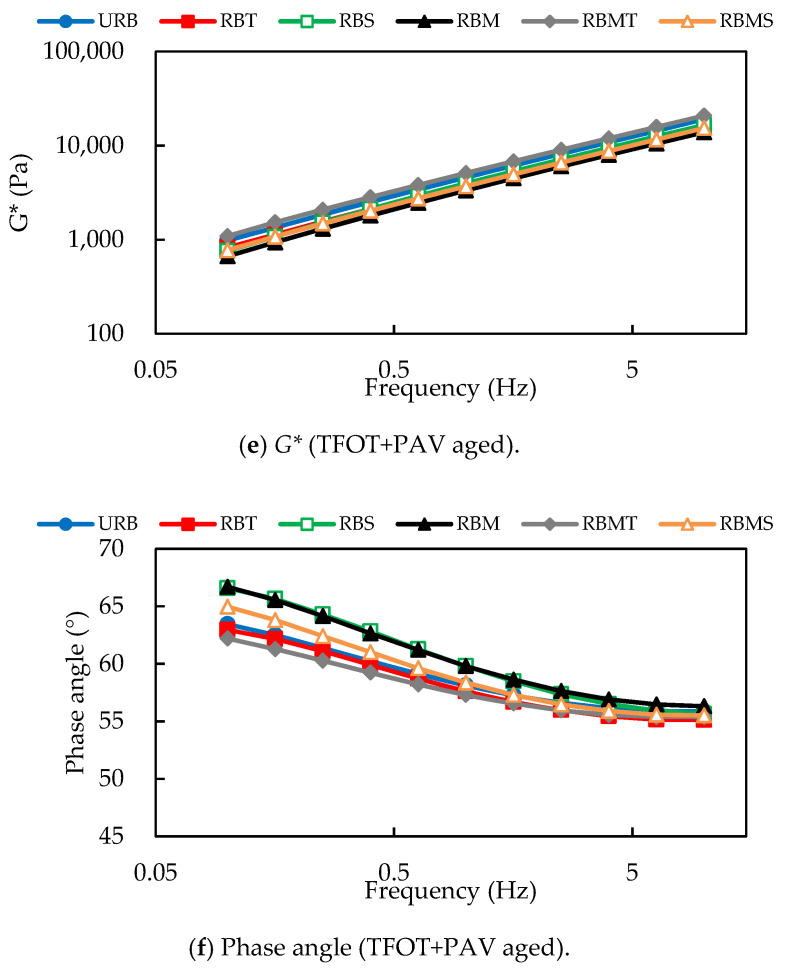
Effect of modification methods on the complex modulus and phase angles of rubberized bitumen depends on frequency sweep test.

As indicated in Figure 11, the phase angles declined smoothly while frequency rose for the unaged binders and binders suffered from STA and LTA, which was mainly because of the existence of CR [50]. It can be observed in Figure 10b that the phase angle of RBT was the lowest and that of RBM was the largest; the orderliness was consistent with the temperature sweep test.

Figure 11c,d presents the phase angles and G* of rubberized bitumen after STA. RBT and RBMT showed lower phase angles than others, and RBT was the lowest. This demonstrated that in the frequency sweep test, TOR has a relatively massive impact on the rheological property after STA. RBM showed the highest phase angle and the lowest G* compared with others. This demonstrated that microwave activation has an adverse effect on the high temperature rheological property after STA.

Figure 11e,f shows the results of phase angle and G* of rubberized bitumen after LTA. It can be seen that at low frequency, the difference of phase angles between different rubberized bitumen was evident, but with the frequency increasing, it was gradually decreasing.

From Figure 11a,c,e, it can be seen that the G* of rubberized bitumen increased as the frequency increased and that it showed the linear dependence of G* with the frequency in the double logarithmic coordinates.

## 5. Conclusions

The main objective of this paper is to evaluate the influence of different modification methods on the aging resistance of rubberized bitumen at an identical preparation process. The preliminary findings are concluded as follows.

(1) TOR and Sasobit improved the high-temperature stability and elastic recoverability of rubberized bitumen, but the effect of Sasobit was barely noticeable. However, microwave-activated rubberized bitumen had an adverse effect on these properties.
(2) STA had a significant effect on the high-temperature stability and elastic recoverability of rubberized bitumen. The TOR-modified rubberized bitumen showed the best aging resistance among the six rubberized bitumens. The CR activated by microwave has worse aging resistance compared with other modification methods. From the temperature sweep test and frequency sweep test, it can be seen that compared with unaged binders, the difference between the rheological properties of various rubberized bitumen after STA was reduced.
(3) Among various modification methods, TOR modifier showed the best aging resistance to LTA, while microwave activation resulted in a weaker aging resistance due to the cracking of CR. From the temperature sweep test and frequency sweep test, it can be seen that the difference between the rheological properties of various bitumen after LTA was further reduced.
(4) This study evaluated the influence of the modification methods on the aging resistance for rubberized bitumen by analyzing the difference in its properties before and after aging. Future research is suggested to investigate the mechanism of different modification methods before and after aging.

## Figures and Tables

**Table 1:** Physical properties of SK-90# bitumen.

Test Items		Unit	Results	Specification
Penetration (25 °C, 100 g, 5 s)		0.1 mm	94.5	T0604
Ductility (15 °C, 5 cm/min)		cm	>150	T0605
Softening point		°C	45.9	T0606
RTFOT aging	Mass change	%	+0.4	T0610
	Residual ductility (10 °C)	cm	12	T0605
	Residual penetration ratio (25 °C)	%	57.8	T0604

**Table 2:** Details and labels of different rubberized bitumen samples.

Samples	Details	Labels
Un-modification rubberized bitumen	CR: 24% by weight of bitumen	URB
Rubberized bitumen modified with TOR	CR: 24% by weight of bitumen TOR: 4.5% by weight of CR	RBT
Rubberized bitumen modified with WMA	CR: 24% by weight of bitumen WMA: 1% by weight of bitumen	RBS
Rubberized bitumen contained microwave pretreated CR	Pre-treated CR: 24% by weight of bitumen	RBM
Rubberized bitumen contained microwave pretreated CR and WMA	Pre-treated CR: 24% by weight of bitumen WMA: 1% by weight of bitumen	RBMS
Rubberized bitumen contained microwave pretreated CR and TOR	Pre-treated CR: 24% by weight of bitumen TOR: 4.5% by weight of CR	RBMT

**Table 3:** Technological tests, normative standards, and details performed on bitumen samples.

Test	Standard	Details	
Softening point	ASTM D36	-	
Elastic recovery	ASTM D6084	Temperature: 25 °C	
Force-ductility	-	Temperature: 5 °C	
Temperature sweep	ASTM D 7175	Plates diameter: 25 mm Gap: 1 mm	Temperature: 30~80 °C Frequency: 0.1 Hz
Frequency sweep			Temperature: 60 °C Frequency: 0.1~10 Hz
